# Centralized Telephone Bureau for Physicians

**Published:** 1916-07

**Authors:** 


### Centralized Telephone Bureau for Physicians.

In some cities where physicians congregate in office buildings, messages are transmitted through a switchboard and the attention to telephones during the physician's absence is economically cared for. In Denver, a private enterprise has recently taken up this work. Cards are distributed bearing the legend "If you cannot find your physician call No. ——" In the early days of the independent service in another city, all instruments were equipped with a pointer which showed that a call had been missed and a special operator took the numbers calling in vain. Edison has invented a phonographic attachment but it is not in practical operation, so far as is known. We knew one young physician whose personal acquaintance with operators enabled him to have his telephone watched in his rather frequent and irregular absences from his office.

Would it not be possible to have a notice published in telephone directories and otherwise brought to the attention of the public that by calling a certain number, information as to the whereabouts, office hours, residence apart from office, vacations, etc., of any physician could be obtained and that messages might be left for him? This could be attended to by the telephone company, by a nurses' register, by any medical organization with a home or in some similar way. It would cost something, however managed and it would be worth a reasonable fee. One free service to a physician not enrolled, would probably secure him as a subscriber. If not, it could simply be reported that that physician was not on the list. A card index would easily give the hours, routine and probable places at which a physician could be reached at any time. Temporary memoranda could be made to anticipate calls and, in the case of absence from town, telephone out of order, etc., the caller could learn when and how to reach the physician desired. Of course, any such system to be of general value would have to be absolutely impartial, accommodating and free from graft.

